# Triple-negative breast cancer: recent treatment advances

**DOI:** 10.12688/f1000research.18888.1

**Published:** 2019-08-02

**Authors:** Alice R T Bergin, Sherene Loi

**Affiliations:** 1 Peter MacCallum Cancer Centre, Melbourne, Victoria, Australia; 2 Sir Peter MacCallum Department of Oncology, University of Melbourne, Melbourne, Victoria, Australia

**Keywords:** Triple negative breast cancer, Immunotherapy

## Abstract

Triple-negative breast cancer (TNBC) is a breast cancer subtype renowned for its capacity to affect younger women, metastasise early despite optimal adjuvant treatment and carry a poor prognosis. Neoadjuvant therapy has focused on combinations of systemic agents to optimise pathological complete response. Treatment algorithms now guide the management of patients with or without residual disease, but metastatic TNBC continues to harbour a poor prognosis. Innovative, multi-drug combination systemic therapies in the neoadjuvant and adjuvant settings have led to significant improvements in outcomes, particularly over the past decade. Recently published advances in the treatment of metastatic TNBC have shown impressive results with poly (ADP-ribose) polymerase (PARP) inhibitors and immunotherapy agents. Immunotherapy agents in combination with traditional systemic chemotherapy have been shown to alter the natural history of this devastating condition, particularly in patients whose tumours are positive for programmed cell death ligand 1 (PD-L1).

## Introduction

Triple-negative breast cancer (TNBC) is a molecularly diverse ^1^ breast cancer subtype currently defined by what it lacks. With hormone receptor immunohistochemistry (IHC) stains of less than 1% for oestrogen and progesterone ^2^ and the absence of HER2 protein overexpression or *HER2* gene amplification (or both) ^3^, TNBC accounts for 12 to 17% of all breast cancers, typically affects younger women and typically carries a poor prognosis ^4^. Metastatic progression in this phenotype is typically marked by early relapse and a predominance of hepatic, pulmonary and central nervous system metastasis ^5^.

Despite, or perhaps because of, its aggressive nature and the lack of current targeted treatments, significant clinical and laboratory research is providing nuanced treatment options. Historically, chemotherapy has been the only viable systemic treatment option for early and advanced disease. However, recently published clinical trials have shown that immunotherapy has an important role in the treatment paradigm of this devastating condition.

## Neoadjuvant chemotherapy for early-stage disease and optimising rates of pathological complete response

Although it is generally accepted that early-stage TNBC is chemotherapy-sensitive, the optimal treatment regimen remains undefined. Neoadjuvant chemotherapy is a standard of care for a locally advanced or inoperable TNBC. A major advantage of this approach is the ability to pre-emptively predict survival according to the presence or absence of a pathological complete response (pCR) at the time of surgery and tailor adjuvant therapy. Patients with TNBC, as opposed to those with the luminal subtypes, are more likely to achieve a pCR with neoadjuvant chemotherapy ^6^. Achieving pCR (defined as no invasive or *in situ* disease in the breast or lymph nodes) at the time of surgery is associated with a significant improvement in disease-free survival (DFS) ^7– 9^; as such, pCR is considered a surrogate outcome end point. However, it is unclear whether changes in pCR will ultimately equate to improvements in overall survival (OS) and thus the use of pCR as a robust trial end point is debated.

Clinicians often adopt an intensive approach with sequential anthracycline and taxane regimens and the evidence for this derives from retrospective, subgroup analyses of clinical trials reported before 2010 ( Table 1).

**Table 1.** Neoadjuvant breast cancer clinical trials pre-2010, including patients with triple-negative breast cancer and showing modest pathological complete response rates with combinations of chemotherapy.

Number of patients with triple-negative breast cancer	Trial arms (number of patients)	Pathological complete response rate	Reference
96	Intensified FAC (56) FEC (40)	Intensified FAC: 47% FEC: 13% Combined: 29%	10
120	FAC or FEC	17%	11
22	T-FAC	45%	19
23	Anthracycline and taxane	39%	12
34	AC ± taxane	27%	13
47	D and A	17%	14
255	A: FAC or FEC or AC (70) B: T-FAC or T-FEC (125) C: Taxane only (17) D: Other (43)	A: 20% B: 28% C: 12% D: 14%	6
45	AC → T	34%	15
21	Anthracycline and taxane	38%	16
38	AC or AT Or T/cape	34%	17
22	Cis	23%	20
12	Cp and T	67%	21
30	E/Cis/F → T	40%	22
125	Platinum and D ± AC	34%	23
10	Cis	90%	24

A, doxorubicin; AC, doxorubicin and cyclophosphamide; Cape, capecitabine; Cis, cisplatin; Cp, carboplatin; D, docetaxel; E/Cis/F, epirubicin and cisplatin and 5-fluorouracil; FAC, 5-fluorouracil and doxorubicin and cyclophosphamide; FEC, 5-fluorouracil and epirubicin and cyclophosphamide; T, paclitaxel.

Anthracyclines alone had reported pCR rates of 14 to 47% ^10, 11^, whereas sequential anthracycline and taxane regimens had reported pCR rates of 17 to 39% ^6, 12– 17^. GeparTrio reported pCR rates up to 57% for TNBC managed with neoadjuvant anthracyclines, cyclophosphamide and taxanes ^18^. Since then, clinical trials have attempted to define which combination of systemic agents results in the highest rates of pCR ( Table 2).

**Table 2.** Neoadjuvant triple-negative breast cancer clinical trials post-2010 showing pathological complete response rates with combinations of chemotherapy, PARP inhibitors and novel agents.

Study Phase ClinicalTrials.gov Identifier	Number of patients	Trial arms	Pathological complete response
**PARP inhibitors**			
BrighTNess ^25^ Phase 3 NCT02032277	A: 316 B: 160 C: 158	A: Veliparib + Cp + T → AC B: Placebo and Cp and T → AC C: Placebo and T → AC.	A: 53% B: 58% C: 31%
Talazoparib ^26^ Phase 2 NCT02282345	17	24 weeks Tala (no neoadjuvant chemotherapy)	47% ^a^
**Anthracycline, taxane and platinum combinations**			
GeparSepto GBG 69 ^27^ Phase 3 NCT01583426	276	Nab-pac → EC Pac → EC	Nab-pac: 56% Pac: 37%
ETNA ^28^ Phase 3 NCT01822314	219	Nab-pac → AC or EC or FEC Pac → AC or EC or FEC	Nab-pac: 41% Pac: 37%
WSG-ADAPT-TN ^29^ Phase 2 NCT01815242	336	Nab-pac and gem Nab-pac and Cp	Nab-pac and gem: 28.7% Nab-pac and Cp: 45.9%
Phase 2 ^30^ NCT01276769	91	T and Cp → surgery → anthracycline EP → surgery → taxane	T and Cp: 38.6% EP: 4%
GEICAM/2006-03 ^31^ NCT00432172	94	EC-D or EC-D and Cp	EC-D: 30% EC-D & Cp: 30%
Cisplatin-1 ^32^ NCT00148694	28	Neoadjuvant cis → surgery → adjuvant chemotherapy	22%
Phase 1 ^33^ NCT01090128	10 (TNBC cohort)	Nab-pac AC	100%
**Chemotherapy backbone with or without novel agents**			
PrECOG 0105 ^34^ Phase 2 NCT00813956	80	Gemcitabine, Cp, iniparib	36%
Cisplatin-2 NCT00580333	51	Cis and Bev	16%
CALGB 40603 ^35^ Phase 2 NCT00861705	454	T ± Cp ± bev → ddAC	No Cp: 41% with Cp: 54% No bev: 52% Bev: 44% Cp and bev: 60%
Phase 2 ^36^ NCT00930930	145	Cis + T ± everolimus	Everolimus: 36% Placebo: 49%
Phase 2 ^37^ NCT00600249	35	Cetuximab and D	pCR: 24%
GeparQuinto GBG 44 ^38^ Phase 3 NCT00567554	663	EC → D ± bev	With bev: 39.3% No bev: 27.9%
Phase 2 ^39^ NCT00933517	47	Panitumumab and FEC-D	46.8%
GeparSixto GBG 66 ^40^ Phase 3 NCT01426880	315 (TNBC cohort)	T and Liposomal doxorubicin and Bev ± Cp	53.2% with Cp 36.9% no Cp

AC, doxorubicin and cyclophosphamide; Bev, bevacizumab; Cis, cisplatin; Cp, carboplatin; D, docetaxel; ddAC, dose dense doxorubicin and cyclophosphamide; EC, epirubicin and cyclophosphamide; EP, epirubicin and paclitaxel; FEC, 5-fluorouracil and epirubicin and cyclophosphamide; gem, gemcitabine; Nab-pac, nab-paclitaxel; pac, paclitaxel; PARP, poly (ADP-ribose) polymerase; T, paclitaxel; Tala, talazoparib; TNBC, triple-negative breast cancer. ^a^Reported as residual cancer burden (RCB) and results represent RCB 0, equivalent to pathological complete response (pCR).

Alkylating agents like carboplatin and cisplatin have provided additional improvements in rates of pCR. Given that a proportion of TNBC tumours have a functional alteration in breast cancer gene 1 ( *BRCA1*), analysis of the role of inter-strand cross-linking agents is especially prudent. The coupling of platinum-induced DNA damage and deficiencies in BRCA-associated DNA repair ^13^ has been exploited in phase 2 trials of platinum monotherapy and yielded promising pCR rates of 23 to 90% ^20, 24, 32^, and rates of pCR were higher amongst BRCA mutation carriers ^24, 32^. Although the randomised phase 2 GEICAM 2006-03 ^31^ did not lead to a significant improvement in pCR, GeparSixto ^40^ and CALGB 40603 ^35^ reported higher rates of pCR with the addition of carboplatin. It is important to note that the addition of carboplatin in these trials led to a significant increase in toxicity and that, for CALGB 40603, the improved pCR rate translated into a modest 5% improvement in 3-year event-free survival, which was not statistically significant ^35^.

In further attempts to manipulate homologous recombination deficiencies inherent to *BRCA1* and *BRCA2* germline mutant tumours, poly (ADP-ribose) polymerase (PARP) inhibitors have been added to the neoadjuvant cocktail. PARP inhibitors act by inducing synthetic lethality in BRCA-deficient cells whilst sparing cells with preserved BRCA function. The phase 3 BrighTNess clinical trial saw a pCR improvement that was attributable to carboplatin rather than the PARP inhibitor under investigation, veliparib ^25^. PrECOG 0105, a single-arm phase 2 clinical trial of gemcitabine, carboplatin and iniparib, yielded a promising pCR of 36%, and response rates were higher in those tumours with elevated mean homologous recombination deficiency-loss of heterozygosity (HRD-LOH) scores, a DNA-based measure of genomic instability ^34, 41^. Although iniparib is no longer considered a true PARP inhibitor ^42– 44^, these results are compelling. It is possible that the different PARP agents will have differing efficacy because of PARP trapping ^45^. Certainly, promising pCR rates were seen in patients with germline BRCA-mutated early-stage breast cancers with just talozparib alone ^26^.

Novel agents like the monoclonal antibodies bevacizumab, panitumumab and cetuximab have been assessed with mixed results ( Table 2). The randomised phase 3 GeparQuinto reported that an improvement was seen in rates of pCR with the addition of bevacizumab, but the survival analysis did not show a significant difference ^38^.

## Managing residual disease following neoadjuvant chemotherapy

Although attaining pCR is the goal of neoadjuvant therapy, optimal management of those who do not meet this end point is critical as these patients have a relapse risk that is six to nine times higher than that of patients achieving pCR ^6, 7^.

The CREATE-X clinical trial showed that six to eight cycles of adjuvant capecitabine (1250 mg/m ^2^ from days 1 to 14, every 21 days) improved DFS and OS in the TNBC cohort. DFS rates were 69.8% in the capecitabine arm and 56.1% in the control arm (hazard ratio [HR] 0.58 for recurrence, second cancer, or death; 95% confidence interval [CI] 0.39–0.87), and OS rates were 78.8% and 70.3% (HR 0.52 for death, 95% CI 0.3–0.9) ^46^. The importance of targeting adjuvant capecitabine to those with residual disease was recently highlighted by the results of the phase 3 GEICAM/CIBOMA trial. This randomised phase 3 trial of 876 patients who had early-stage TNBC and who had completed standard adjuvant or neoadjuvant polychemotherapy was designed to analyse the impact of adjuvant capecitabine (1000 mg/m ^2^ from days 1 to 14, every 21 days) for all patients with TNBC regardless of their pCR status. There was no significant difference in 5-year DFS and OS between the treatment groups, highlighting the need to choose a treatment-resistant group ^47^. The results of the CREATE-X trial now compel most clinicians to treat early-stage TNBC with neoadjuvant chemotherapy in order to understand who should have capecitabine. Whilst capecitabine should be considered, ongoing trials are evaluating new agents for TNBC with residual disease after neoadjuvant chemotherapy.

## Does immunotherapy (CTLA4 and PD-(L)1 inhibitors) improve pathological complete response?

The programmed cell death 1 (PD-1) inhibitors nivolumab and pembrolizumab and the programmed cell death ligand 1 (PD-L1) inhibitor atezolizumab are monoclonal antibodies designed to release inhibition of the PD-1/PD-L1–mediated immune response, whereas ipilimumab releases inhibition of the cytotoxic T-lymphocyte-associated protein 4 (CTLA4)-mediated immune response. TNBC tumour cells use the PD-1/PD-L1 and CTLA4 immune pathways to avoid immune surveillance and proliferate but these monoclonal antibodies facilitate an effective immune-mediated and anti-tumour response ^48^.

Pembrolizumab combined with anthracycline and taxane chemotherapy has pushed the pCR boundary even further. Impressive pCR rates of up to 90% have been reported in phase 1b and 2 clinical trials ( Table 3).

**Table 3.** Neoadjuvant clinical trials in triple-negative breast cancer using combinations of chemotherapy with or without immunotherapy.

Study	Number of patients	Trial arms	pCR rate
I-SPY 2 ^52^ Phase 2	69	T → AC T and pembro → AC	Control: 22.3% Pembro: 62.4%
KEYNOTE-173 ^53, 54^ Phase 1b	20	A: pembro → pembro and nab-pac → pembro and AC. B: pembro → pembro and nab-pac 100 mg/m ^2^ and Cp (AUC 6) → pembro and AC C: pembro → pembro and nab-pac 125 mg/m ^2^ and Cp (AUC 5)→ pembro and AC D: pembro → pembro and nab-pac 125 mg/m ^2^ and Cp (AUC 2)→ pembro and AC E: pembro → pembro and T and Cp (AUC 5)→ pembro and AC F: pembro → pembro and T and Cp (AUC 2)→ pembro and AC	A: 60% B: 90% Overall pCR rate (A–E): 60%

AC, doxorubicin and cyclophosphamide; AUC, area under curve; Cp, carboplatin; Nab-pac, nab-paclitaxel; pCR, pathological complete response; Pembro, pembrolizumab; T, paclitaxel.

Patient selection for the optimal use of these agents is important and will likely be critical to their success in terms of DFS and OS outcomes, as seen in the CREATE-X and GEICAM-CIBOMA trials. The BCT 1702-CHARIOT clinical trial (ANZCTR N12617000651381) was designed to help guide clinicians in the management of patients with TNBC that is not responding to standard neoadjuvant therapy. The phase 2 clinical trial combines paclitaxel with ipilimumab and nivolumab in eligible patients with a residual TNBC of at least 15 mm and less than 50% reduction in longest diameter of the tumour after completion of four standard cycles of anthracycline chemotherapy. The trial is designed to select out the most at-risk TNBC population to see whether they can derive benefit from the novel combination of therapies as these patients have been reported to have pCR rates of less than 10% and hence the highest risk of dying from their disease ^49, 50^. Furthermore, selection and duration of these myriad adjuvant therapies will be important to delineate as the outcomes of ongoing clinical trials ( Table 4) are eagerly awaited.

**Table 4.** Ongoing, unreported phase 3 clinical trials of (neo)adjuvant chemotherapy with or without immunotherapy.

Study	Agents/Intervention	Outcome of interest
**Neoadjuvant studies**		
Impassion031 NCT03197935	Atezolizumab Nab-paclitaxel Anthracyclines	pCR EFS OS
NeoTRIPaPDL1 NCT02620280	Atezolizumab Carboplatin Abraxane AC, EC or FEC	EFS pCR
Keynote522 NCT03036488	Pembrolizumab Paclitaxel, carboplatin Anthracycline	pCR EFS OS
**Adjuvant studies**		
SWOG 1418 NCT02954874	Pembrolizumab	iDFS OS dRFS
IMpassion030 NCT03498716	Atezolizumab Paclitaxel ddAC or ddEC	iDFS OS DFS RFI
A-Brave NCT02926196	Avelumab	DFS OS

AC, doxorubicin and cyclophosphamide; ddAC, dose dense doxorubicin and cyclophosphamide; ddEC, dose dense epirubicin and cyclophosphamide; DFS, disease-free survival; dRFS, disease recurrence-free survival; EC, epirubicin and cyclophosphamide; EFS, event-free survival; FEC, 5-fluorouracil and epirubicin and cyclophosphamide; iDFS, invasive disease-free survival; OS, overall survival; pCR, pathological complete response; RFI, recurrence-free interval.

## Systemic therapy for metastatic disease

Patients with metastatic TNBC experience poorer outcomes when compared with patients with other breast cancer subtypes ^51^. First-line systemic treatment typically includes a taxane or anthracycline combination ^55^, and median OS tends to be 18 months or less ^56– 58^. Novel treatment approaches are critical to improve these dire survival outcomes.

## Role of poly (ADP-ribose) polymerase inhibitors and chemotherapy for *BRCA*1 and *BRCA*2 mutation carriers

The OlympiAD clinical trial randomly assigned patients with advanced HER2-negative breast cancer and a germline *BRCA* mutation to a PARP inhibitor, olaparib (300 mg twice daily), or standard physician’s choice chemotherapy ^59^. The significant progression-free survival (PFS) benefit favoured olaparib with a median PFS of 7.2 months (versus 4.2 months) ^59^. Subgroup analysis of PFS for randomised stratification factors revealed an outstanding HR for progression of 0.39 (95% CI 0.27–0.57) amongst the TNBC subset, which made up nearly 50% of the treatment cohorts in both arms ^59^.

The EMBRACA clinical trial compared the PARP inhibitor talazoparib (1 mg daily) with protocol-specified standard therapy (capecitabine, eribulin, gemcitabine or vinorelbine) and found a favourable median PFS of 8.6 versus 5.6 months in the standard therapy group (HR for progression or death 0.54, 95% CI 0.41–0.71) with a trend towards an OS benefit, but the data are immature ^60^. Although rates of adverse events were similar in the two treatment arms, patients randomly assigned to talazoparib reported superior quality-of-life outcomes (as recorded by the EORTC QLQ-C30) with a significant delay in the onset of a clinically meaningful deterioration in global health status ^60^.

The results of the randomised phase 3 trials BRAVO (ClinicalTrials.gov Identifier: NCT01905592) using niraparib 300 mg daily ^61^ versus chemotherapy and BROCADE (ClinicalTrials.gov Identifier: NCT02163694) using veliparib or placebo combined with chemotherapy in a similar cohort (germline *BRCA* mutation-positive) are still pending.

The addition of iniparib to gemcitabine and carboplatin has shown promising results for all patients with metastatic TNBC regardless of their *BRCA* mutation status. A randomised phase 2 clinical trial showed that the addition of iniparib prolonged the median PFS from 3.6 to 5.9 months (HR for progression, 0.59; *P* = 0.01) and the median OS from 7.7 to 12.3 months (HR for death, 0.57; *P* = 0.01) ^62^. The phase 3 clinical trial did not meet the pre-specified co-primary end points, PFS and OS, but did report an efficacy signal for patients randomly assigned to second- or third-line PARP inhibitor therapy ^62^. This is likely because iniparib is no longer considered a true PARP inhibitor for the purposes of clinical research. Although iniparib inhibited PARP-1 function *in vitro* and was tested in clinical trials for this reason, subsequent studies have shown that the cell killing mechanism of iniparib does not reflect PARP inhibition ^42– 44^.

Notably, the Triple-Negative Breast Cancer Trial (TNT) has provided important insights into the role of platinum- and taxane-based chemotherapy ^63^. The trial enrolled 376 patients with either a known deleterious *BRCA1/2* germline mutation (and any metastatic breast cancer phenotype) or metastatic TNBC. Although no significant difference was seen in the overall TNT population, a significantly better objective response rate of 68% to carboplatin versus 33% to docetaxel was found amongst the 43 patients with a germline *BRCA1/2* mutation ^63^. Furthermore, within this population, a PFS benefit favouring carboplatin (median PFS of 6.8 versus 4.4 months) was found without a corresponding OS benefit ^63^. Once again, the benefit was not reflected in the overall TNT population, where there was no significant PFS or OS advantage to either agent ^63^.

## IMpassion 130: Will immunotherapy be the winner for advanced triple-negative breast cancer too?

Prior to October 2018, phase 1 and 2 clinical trials evaluating PD-1 protein blockade as monotherapy in advanced TNBC showed disappointing response rates of 5 to 10% in unselected cohorts ^64– 66^. These poor response rates likely reflect that breast cancer is not a highly immunogenic solid organ malignancy ^67^. This has been thought to underlie the modest response rates seen with checkpoint inhibitor monotherapy to date; as a result, patients with advanced breast cancer need to be selected for the presence of pre-existing activity of the host immune system ^68, 69^. The complexity of this response, when analysed in more detail, is apparent; however, tumour-infiltrating lymphocytes (TILs) simply measured by using light microscopy on hematoxylin-and-eosin–stained slides particularly have provided important insights into this variable response rate ^70^. TILs are mononuclear immune cells that infiltrate tumour tissue and are composed mainly of CD4 ^+^ and CD8 ^+^ (cytotoxic) T cells ^71^. TILs are an independent prognostic biomarker in breast cancer, and in early-stage, node-positive TNBC, high TILs correlatewith improved survival ^72, 73^. In addition to TILs, PD-1 and PD-L1 can be expressed by tumour cells and their presence can be evaluated as part of a detailed pathological examination of the tumour by using proprietary IHC assays ^74, 75^. In metastatic TNBC, better response rates were noted with pembrolizumab monotherapy in tumours with higher quantitative levels of TILs ^76^. Ultimately, it is highly likely that all of these immune markers read out a similar signal for selecting patients most likely to respond to PD-1 or PD-L1 inhibition or both.

The recent approval of atezolizumab in advanced TNBC was based on the IMpassion 130 study. IMpassion 130 was a phase 3 registration study that randomly assigned over 900 patients with incurable TNBC who had relapsed 12 months or more after adjuvant chemotherapy to receive either nab-paclitaxel and atezolizumab (a PD-L1 inhibitor) or nab-paclitaxel and placebo. A statistically superior PFS benefit was seen: median PFS values of 7.2 months (95% CI 5.6–7.2 months) in the atezolizumab and taxane arm and 5.5 months (95% CI 5.3–5.6 months) with chemotherapy alone (HR = 0.8, 95% CI 0.69–0.92; *P* = 0.0025) were reported; among the PD-L1–positive tumours, median PFS values of 7.5 months (95% CI 6.7–9.2 months) and 5 months (95% CI 3.8–5.6 months) were reported (HR = 0.62, 95% CI 0.49–0.78; *P* <0.0001); hence, the primary end point of the study was met ^77^. Interim OS analysis (60% of events) already showed a trend towards the atezolizumab and taxane combination with median OS values of 21.3 and 17.6 months (stratified HR 0.84, 95% CI 0.69–1.02) ^77^. Furthermore, 40% of the population did not receive any prior chemotherapy. It is likely that this group of patients with metastatic TNBC does much better both in general and with immunotherapy. Still, the first steps have been taken in the field, and we have much work to do to positively impact survival in this population.

## What can we expect next?

Recently, the treatment of both early and advanced TNBC has seen significant improvements in response rates and survival outcomes. The time has now come to stratify and personalise patient management according to response for early-stage disease and to the presence or absence of an immune infiltrate for advanced disease.

Patients with early-stage disease who do not achieve pCR after neoadjuvant chemotherapy should be offered six to eight cycles of adjuvant capecitabine monotherapy, in accordance with the CREATE-X trial. For patients with advanced disease who are PD-L1 ^+^, CD8 ^+^, or TIL ^+^, optimal treatment would include up-front atezolizumab and nab-paclitaxel. Exposure to a PD-1 or PD-L1 agent (or both) is likely still important for survival in patients who do not receive the combination in the first-line setting. Whether those who have a positive immune infiltrate and a disease-free interval of less than 12 months benefit from this regimen is unknown. Those without a positive immune infiltrate should be referred for a clinical trial that uses combinations of novel agents, chemotherapy and immunotherapy. The TNBC treatment landscape is an ever-evolving space, which epitomises the crucial relationship between laboratory and clinical research. The complex interplay has enabled practise-changing advances in treatment outcomes not seen in TNBC for decades.

## Abbreviations

BRCA, breast cancer gene; DFS, disease-free survival; OS, overall survival; PARP, poly (ADP-ribose) polymerase; pCR, pathological complete response; PD-1, programmed cell death 1; PD-L1, programmed cell death ligand 1; PFS, progression-free survival; TIL, tumour-infiltrating lymphocyte; TNBC, triple-negative breast cancer
